# Treatment Response and Safety Profile of Nintedanib in Connective Tissue Disease-Associated Interstitial Lung Disease: A Retrospective Observational Study

**DOI:** 10.31138/mjr.080825.lfh

**Published:** 2025-09-30

**Authors:** Arriana Gkouvi, Maria Boutel, Nikoleta Zioga, Eleni Pagkopoulou, Maria Mytilinaiou, Christina Rampiadou, George A. Margaritopoulos, Dimitrios P. Bogdanos, Theodoros Dimitroulas, Theodora Simopoulou

**Affiliations:** 1 Department of Rheumatology and Clinical Immunology, Faculty of Medicine, School of Health Sciences, University of Thessaly, University General Hospital of Larissa, Larissa, Greece;; 2 Fourth Department of Internal Medicine, Hippokration University Hospital, Medical School, Aristotle University of Thessaloniki, Thessaloniki, Greece;; 3 Department of Respiratory Medicine, G. Papanikolaou Hospital, Thessaloniki, Greece;; 4 Interstitial Lung Disease Unit, Respiratory Medicine Department, G. Papanikolaou Hospital, Medical School, Aristotle University of Thessaloniki, Thessaloniki, Greece

**Keywords:** nintedanib, CTD-ILD, SSc-ILD, antifibrotic, efficacy, safety

## Abstract

**Objective:**

Interstitial lung disease (ILD) is a significant clinical complication that can occur in various connective tissue diseases (CTDs). Treatment includes immunosuppressants to reduce inflammation, while the antifibrotic nintedanib has been approved for slowing lung function decline in certain CTD-ILD patients. We aimed to examine the use of nintedanib in a Greek population, offering insight in real-world clinical setting.

**Methods:**

Retrospective collection of data from patients with various CTD-ILDs receiving nintedanib, including demographic, clinical and laboratory features. The analysis comprised records of pulmonary function tests (PFTs) conducted at all available time points prior to and following treatment with nintedanib, in addition to assessments of the drug’s tolerability and the incidence of significant adverse events.

**Results:**

A total of 60 patients with CTD-ILD (43 females, mean age 60.1 ± 11.8, 50% systemic sclerosis, 21.7% rheumatoid arthritis, 13.3% idiopathic inflammatory myopathies) were included. 58 patients (96.6%) received nintedanib in combination with immunosuppressants. The mean baseline FVC% was 66.5 ± 16.4 and the mean FVC% after treatment was 64.1 ± 19.0, showing a downward trend (p=0.090). The presence of other systemic manifestations such as PAH, cardiac involvement, digital ulcers or GI manifestations emerged as a significant predictor in both %change and absolute DLCO difference. Dose reduction occurred in 16 (26.7%) patients while permanent discontinuation only in four patients (6.7%). The most common adverse event was diarrhoea (21.6%).

**Conclusion:**

Our real-world data across a broad spectrum of CTD-ILD suggests that nintedanib is associated with disease stabilisation, and is generally well tolerated. It may be beneficial in combination with immunosuppressives in slowing the rate of lung function decline.

## INTRODUCTION

Interstitial lung disease (ILD) is a clinically significant manifestation occurring in the context of every connective tissue disease (CTD), collectively referred to as CTD-ILD.^1^ The diseases with the highest prevalence of ILD are systemic sclerosis (SSc), rheumatoid arthritis (RA), mixed connective tissue disease (MCTD), and idiopathic inflammatory myopathies (IIM).^1^

Despite being present in different clinical entities, CTD-ILDs share similarities in the underlying pathophysiology attributed to immune-mediated inflammation and fibrogenesis. The disease course varies widely; some patients experience slow progression or long-lasting steady disease, while others develop rapidly progressive ILD with poor prognosis and high mortality.^2^

Occasionally, ILD is the first feature of an underlying autoimmune disease, while in most cases it develops in the course of the disease.^3^ The diagnosis of CTD-ILD is based on a combination of clinical evaluation, pulmonary function tests (PFTs), and high-resolution computed tomography (HRCT) of the chest.^4^ Clinically, patients often present with progressive exertional dyspnoea and a nonproductive cough, along with systemic signs and symptoms suggestive of the underlying CTD, such as arthralgia/arthritis, Raynaud’s, skin thickening, rash or muscle weakness. Physical examination may reveal fine ‘’velcro’’ sounds at the lower lobes. Serologic immunological testing plays a key role in identifying the underlying CTD, with common positive autoantibodies including antinuclear antibodies (ANAs), rheumatoid factor (RF), anti-CCP, anti-Scl-70, anti-Jo-1, anti-RNP, and SSA/SSB, depending on the specific CTD. PFTs typically show a restrictive pattern with reduced forced vital capacity (FVC) and total lung capacity (TLC), along with a decreased diffusing capacity for carbon monoxide (DLCO). HRCT is critical in confirming ILD and identifying specific radiologic patterns. The most frequently observed radiological pattern is that of non-specific interstitial pneumonia (NSIP), seen in most CTD-ILDs except RA, in which the pattern of usual interstitial pneumonia (UIP) prevails.^1,4^ Management of CTD-ILD typically includes immunosuppressive agents such as mycophenolate mofetil (MMF), rituximab, or cyclophosphamide, aiming to mitigate inflammation and slow disease progression.^5^ Glucocorticoids are only used during acute exacerbations, while long-term use is limited.^5^ More recently nintedanib -an oral tyrosine-kinase inhibitor- has demonstrated efficacy in halting pulmonary function decline in patients with SSc-ILD and in patients with progressive fibrosing CTD-ILD defined as a relative decline in the FVC of at least 10% of the predicted value, a relative decline in the FVC of 5% to less than 10% of the predicted value and worsening of respiratory symptoms or an increased extent of fibrosis on HRCT, or worsening of respiratory symptoms and an increased extent of fibrosis.^6,7^ Nintedanib targets vascular endothelial growth factor receptors (VEGF) 1, 2, and 3, platelet-derived growth factor (PDGF) α and β receptors, and fibroblast growth factor receptor (FGF) 1, 2 and 3.^8–10^ By blocking these pathways, nintedanib reduces fibroblast proliferation, differentiation, and extracellular matrix deposition, all of which are integral to the pathogenesis of lung fibrosis.^8–10^

In the modern treatment era combination therapy including immunosuppressives and antifibrotics is strongly recommended for the management of CTDILD.^5,11^ Accumulating real world data in CTD-ILD beyond SSc-ILD is important for guiding clinical practice.^12^ The aim of the current retrospective study was to investigate the use of nintedanib in a Greek population, offering insight in its real-world clinical effectiveness and safety profile in patients with CTD-ILD.

## MATERIALS AND METHODS

### Patients and data collection

We retrospectively collected data from patients with CTD-ILD who were receiving nintedanib and were followed up by the Rheumatology Department of the Fourth Department of Internal Medicine, Hippokration General Hospital, Thessaloniki, Greece and the Department of Rheumatology and Clinical Immunology, University General Hospital of Larissa, Greece, from June 2019 to June 2025. This study follows the STROBE (Strengthening the Reporting of Observational Studies in Epidemiology) guidelines for observational studies. All data was handled anonymously, and due to the retrospective observational nature of this study, ethics approval was not required. Eligible participants met the following criteria: Diagnosis of SSc, RA, IIM or other CTDs according to established criteria (ACR/EULAR) from experienced rheumatologists,^13–15^ diagnosis of ILD via a combination of HRCT, PFTs and multidisciplinary team consensus, nintedanib initiation, and availability of PFT data. Patients with coexisting obstructive pulmonary disease (COPD), previous treatment with pirfenidone or currently on investigative products were excluded from the analysis. Follow-up was conducted according to real world clinical practice and consequently both the number of visits and the intervals between them varied among patients. All patients were treated with nintedanib at a dose of 150mg twice daily (BD) or 100mg BD, depending on individual tolerance and clinical judgement.

We extracted data from medical records using a standardised extraction form (M.B, N.Z), including demographic characteristics (age, gender), duration of CTDILD and type of CTD (SSc, RA, IIM, Undifferentiated Connective Tissue Disease (UCTD), MCTD, Interstitial Pneumonia with Autoimmune Features (IPAF), Juvenile Idiopathic Arthritis (JIA), other diseases). Furthermore, data regarding immunosuppressive medication, systemic manifestations such as gastrointestinal involvement (GI), cardiac involvement or pulmonary arterial hypertension (PAH) and autoantibody profile (Scl-70, RF, Jo-1, others) were extracted. PFTs and specifically FVC (% of the predicted value), FEV1 (% of the predicted value) and DLCO (% of the predictive value) were recorded at all available points before and after treatment with nintedanib. The FVC/DLCO ratio was calculated at baseline and after treatment. For all parameters (FVC%, FEV1%, DLCO%) absolute changes (last value available minus baseline) and relative changes (percentage changes relative to baseline) were calculated to assess individual changes over time. All PFT data was extracted from standardised spirometry reports conducted in accredited laboratories following ERS/ATS guidelines and results were interpreted by pulmonologists.^16^ Additionally, data regarding inflammatory markers [C-reactive protein (CRP) and erythrocyte sedimentation rate (ESR)] were extracted from routine blood tests during clinical visits. Data regarding treatment with nintedanib included the initial dosage, tolerability, as well as continuation or dose reduction or temporary or permanent discontinuation and reason (if applicable). Finally, adverse events (AE) and deaths were recorded.

### Statistical analysis

All data was handled anonymously, and descriptive statistics were calculated for all demographic and disease specific variables. Continuous variables are reported as mean and standard deviation if normally distributed. Otherwise, median and interquartile range (IQR) are also reported. The normality of the continuous variables was assessed with the Shapiro–Wilk test.

For PFTs we calculated absolute differences from the last available value to baseline and relative differences (% changes relative to baseline). Missing data were handled using complete case analysis. Patients with missing values in either baseline measurements or last available PFTs were excluded from the respective analyses. Comparisons were performed using paired t-tests for normally distributed data or the Wilcoxon signed-rank test when normality assumptions were not met. This approach was chosen to evaluate within-subject changes over time. Individual patient trajectories were visualised with line plots connecting baseline and after treatment measurements. Additionally, PFT trajectories over time are depicted for each individual patient.

To explore associations between clinical variables and PFT outcomes univariate linear regression models were used. Both continuous (age, CTD-ILD duration) and categorical variables (gender, systemic manifestations, MMF, corticosteroid or rituximab treatment) were included as independent variables in our regression models. The individual variables from the univariate analyses with p<0.2 were further explored with multivariate linear regression models to identify independent predictors of relative or absolute change in PFTs. No correction for multiple comparisons was applied, as univariate analyses were exploratory and intended to guide the selection of variables for the multivariate model. Statistical analyses were performed using RStudio software [version 4.3.2 (2023-10-31), R Foundation for Statistical Computing, Vienna, Austria] and the SPSS statistical package v27. A p-value < 0.05 was considered statistically significant.

## RESULTS

### Demographic characteristics

During the study period, 60 patients with CTD-ILD receiving nintedanib were identified. All of them met inclusion and exclusion criteria and were consequently included in the study. The mean age was 60.1 ± 11.8 (Median 62, IQR =12), and most patients were female (71.7%). The mean duration of CTD-ILD was 6.7 ± 4.2 years (Median 6, IQR =5), and CTD diagnoses included SSc (50%), RA (21.7%), IIM (13.3%), MCTD/UCTD (8.3%), IPAF (5.0%), and JIA (1.7%). Extrapulmonary manifestations were identified in 56.7% of our patients, and the main types included GI manifestations (44.1%), PAH (23.5%), cardiac involvement (20.6%), or digital ulcers (20.6%). Additionally, 43.3% of our cohort were positive for anti-Scl70 autoantibodies and 16.6% for RF. The median follow-up of our patients was 17.5 months. Four patients (6.6%) developed lung cancer, and one female patient developed breast cancer. Details about baseline characteristics of our sample can be found in **Table 1**, and concomitant treatments that 96.6% of patients received can be found in **Table 2.** The most common immunosuppressive agents received while on nintedanib were glucocorticoids (60.0%), MMF (48.3%) and rituximab (35.0%). As far as other medications are concerned, 83.3% of patients were on proton pump inhibitors, 33.3% on calcium channel blockers and 20.0% on endothelin-1 receptor inhibitors.

**Table 1.** Baseline characteristics of our sample (N=60).

**Parameter**		**Value**
Age	Mean ± SD [Median (IQR)]	60.1 ± 11.8 [62 (12)]
Gender	Male % (n)	28.3% (17)
Disease type	SSc % (n)	50.0% (30)
	RA % (n)	21.7% (13)
	IIM % (n)	13.3% (8)
	IPAF % (n)	5.0% (3)
	MCTD/UCTD % (n)	8.3% (5)
	JIA % (n)	1.7% (1)
CTD-ILD duration (yrs)	Mean ± SD [Median (IQR)]	6.7 ± 4.2 [6 (5)]
Other systemic manifestations		56.7% (34)
Systemic manifestations type	GI % (n)	44.1% (15)
	PAH % (n)	23.5% (8)
	Cardiac involvement % (n)	20.6% (7)
	Ulcers % (n)	20.6% (7)
	Severe skin disease % (n)	8.8% (3)
	CNS % (n)	2.9% (1)
	Nephropathy % (n)	2.9% (1)
Cancer	Lung	4
	Breast	1
Autoantibody profile	anti-Scl70 % (n)	43.3% (26)
	RF % (n)	16.6% (10)
	Others % (n)	35.0% (21)

CNS: Central nervous system; GI: Gastrointestinal; JIA: Juvenile idiopathic arthritis; IIM: idiopathic inflammatory myopathies; IPAF: Interstitial pneumonia with autoimmune features; IQR: Interquartile range; MCTD: mixed connective tissue disease; PAH: Pulmonary arterial hypertension; RA: Rheumatoid arthritis; RF: Rheumatoid factor; SD: Standard deviation; SSc: Systemic sclerosis; UCTD: Undifferentiated connective tissue disease; yrs: years. For continuous variables additional median and IQR are reported when data is not normally distributed.

**Table 2.** Concomitant treatments.

**Treatment**	**N of patients**	**Value (%)**
Prednisolone	36	60.0
Proton pump inhibitors	50	83.3
Mycophenolate mofetil	29	48.3
Rituximab	21	35.0
Hydroxychloroquine	9	15.0
Methotrexate	5	8.3
Tocilizumab	8	13.3
Calcium channel blockers	20	33.3
Endothelin-1 receptor inhibitors	12	20.0
Sildenafil	4	6.7
Intravenous immunoglobulin	3	5.0
Azathioprine	1	1.7
Leflunomide	3	5
Teprostinil	3	5
Epoprostenol	1	1.7
Beta-blocker	4	6.7

### Pulmonary function tests

**Figure 1.**
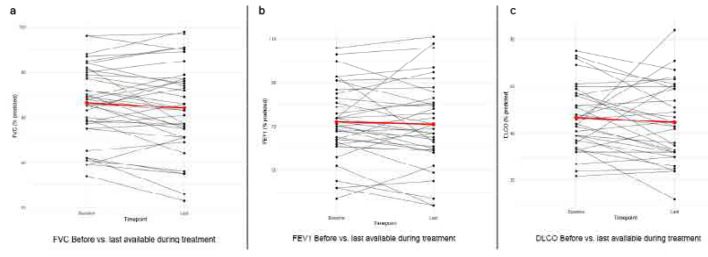
Line plots visualising individual patient PFTs before and after treatment.

**Figure 2.**
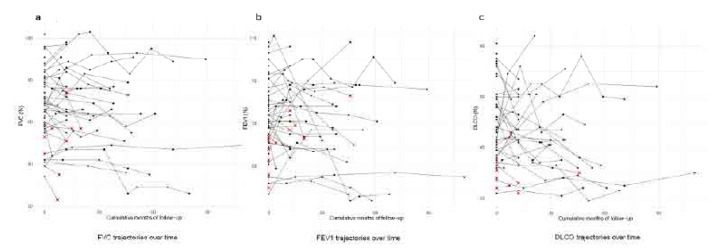
Patient trajectories showing individual values for all patients across available timepoints, showcasing mostly stability of PFTs.

The mean baseline FVC% was 66.5 ± 16.4, and the mean FVC% after treatment was 64.1 ± 19.0, showing a non-significant downward trend (p=0.090, 95% CI: −5.33–0.41), with a relative change in FVC% of −4.2 ± 15% and an absolute change of −2.5 ± 8.8 mainly indicating stability. Similarly, the mean baseline DLCO was 46.8 ± 14.0 and the mean DLCO after treatment was 44.6 ± 16.6 (p=0.14) with a relative change of −2.9 ± 31.9 % and an absolute change of −2.2 ± 14. In the same way, the mean FEV1% was 72.1 ± 16.9 at baseline and decreased non-significantly to 70.9 ± 19.3 after treatment (p=0.398). Similarly, FVC/DLCO mainly remained unchanged after treatment. These results indicate that PFTs remain stable after nintedanib initiation. PFT values are described in detail in **Table 3**. Line plots were generated to visualise individual patient PFTs before and after treatment and can be found in **Figure 1**. Additionally, we created patient trajectories showing individual values for all patients across available time-points, showcasing mostly stability of PFTs (**Figure 2**). All patients had available baseline FVC measurements while one patient did not have available baseline FEV1 and six patients lacked baseline DLCO. Among those with available baseline values, follow-up FVC was missing in 21 patients, FEV1 in 22 patients and DLCO in 21 patients. Details regarding the number of patients contributing to each PFT can be found in **Table 3**.

**Table 3.** Values before and after treatment with nintedanib.

**Variable**	**Characteristics**	**Value**	**N**	**p-value (95% CIs)**
FVC	Baseline FVC	66.5 ± 16.4^†^	39	0.090^#^ (−5.33, 0.41)
	FVC after	64.1 ± 19.0^†^		
	% change in FVC	−4.2 ± 15.0%^†^		
DLCO	Baseline DLCO	46.8 ± 14.0^†^	33	0.139^$^
	DLCO after	44.6 ± 16.6^†^		
	% change in DLCO	−2.9 ± 31.9% [−9.6 (30.3)%] ^††^		
FEV1	Baseline FEV1	72.1 ± 16.9^†^	37	0.398^$^
	FEV1 after	70.9 ± 19.3^†^		
	% change in FEV1	−1.2 ± 16.9%^†^		
FVC/DLCO	Baseline FVC/DLCO	1.6 ± 0.8 [1.5 (0.5)]^††^	31	0.518^$^
	FVC/DLCO after	1.4 ± 0.9 [1.4 (0.7)]^††^		
CRP (mg/dL)	Baseline CRP	1.0 ± 1.6 [0.5 (0.8)]^††^	33	0.623^$^
	CRP after	0.9 ± 1.0 [0.5 (0.8)]^††^		
ESR (mm/hr)	Baseline ESR	25 ± 20.3^†^	28	0.201^#^ (−2.9, 13.2)
	ESR after	30.1 ± 29.6^†^		

†: Mean and standard deviation, data is normally distributed;

††: Additionally median and IQR are reported, data is not normally distributed;

#: paired t test;

$: Wilcoxon signed rank test. CI: Confidence intervals; CRP: C-reactive protein; DLCO: Diffusion capacity for carbon monoxide; ESR: Erythrocyte sedimentation rate; FVC: Forced vital capacity; FEV1: forced expiratory volume in 1 second; IQR: Interquartile range.

### Inflammatory markers

**Figure 3.**
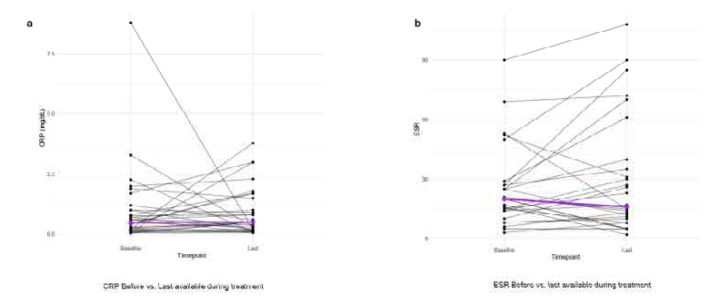
CRP and ESR line plots.

Both CRP and ESR remained mostly unchanged, as expected, since nintedanib is not considered an anti-inflammatory treatment. CRP before treatment initiation was 1.0 ± 1.6 mg/dL (Median 0.5, IQR=0.8) and 0.9 ± 1.0 mg/dL (Median 0.5, IQR=0.8) after treatment (p=0.623) and the corresponding line plots can be found in **Figure 3.** The inflammatory markers’ values can be found in **Table 3.**

### Adverse events

Concerning the adverse effects, 26.7% of patients received nintedanib at half dose due to intolerance. Most frequent adverse events were related to the GI system, with 21.6% of our patients reporting diarrhoea and 15% reporting nausea. One patient (1.7%) suffered from a myocardial infarction. Four patients (6.7%) discontinued treatment and 12 (20%) died, with exacerbation of ILD being the most frequent reason for death (41.7%). Other causes of death were infections (25.0%), lung cancer related (16.7%), cardiogenic shock (8.3%) and cerebrovascular accident (8.3%).

### Additional analyses

Univariate regression analyses were utilized to further explore the association between PFTs relative or absolute change and potential predictors, including age, gender, CTD-ILD years of diagnosis, other systemic manifestations and treatment with MMF, rituximab, or prednisolone (no, previous, current), since these were the immunosuppressive regimens our patients most often used. Regarding relative DLCO% change, the presence of other systemic manifestations was significantly associated with a greater decline in DLCO% (β=−23.6, 95% CI: −45.1–−2.1, p=0.032, R^2^=0.139), indicating that systemic involvement predicts a more pronounced DLCO deterioration. Gender was not significantly associated with DLCO% change (β=16.9, 95% CI: −9.1–42.9, p=0.19), neither was current MMF use (β=15.5, 95% CI: −8.9–39.9, p=0.21), corticosteroid use (β=−3.8, 95% CI: −37.2–29.7, p=0.82), rituximab use (β=−8.3, 95% CI: −34.0–17.5, p=0.52), or CTD-ILD duration (β=−1.9, 95% CI: −6.7–2.8, p=0.41). Age demonstrated a non-significant trend toward greater DLCO decline with older age (β=−0.79 per year, 95% CI: −1.77–0.18, p=0.11), accounting for 8.1% of the outcome variance.

No significant associations between relative FVC% change and predictors were identified. Specifically, univariate regressions showed no significant relationship between FVC% change and age (β=−0.2, 95% CI: −0.6–0.2, p=0.33), gender (β=−4.6, 95% CI: −16.2–7.0, p=0.42), CTD-ILD years (β=0.23, 95% CI: −1.4–1.8, p=0.77), current MMF (β=4.2, 95% CI: −6.2–14.6, p=0.41), glucocorticoid use (β=7.8, 95% CI: −9.0–24.6, p=0.35), rituximab use (β=3.8, 95% CI: −6.7–14.3, p=0.46) or other systemic manifestations (β=−4.0, 95% CI: −13.9–5.9, p=0.42). Similarly, no significant associations were noted between relative FEV1% change and age (β=−0.2, 95% CI: −0.7–0.2, p=0.30), CTD-ILD years (β=0.3, 95% CI: −1.6–2.1, p=0.78), gender (β=−3.5, 95% CI: −16.7–7.8, p=0.6), other systemic manifestations (β=−6.0, 95% CI: −17.4–5.5, p=0.29) and current MMF (β=0.9, 95% CI: −11.4–13.2, p=0.89), corticosteroids (β=9.3, 95% CI: −12.4–31.0, p=0.39) or rituximab (β=3.7, 95% CI: −8.3–15.6, p=0.53). Finally, no associations between FVC/DLCO and the above predictors were identified. Among all predictors tested, only the presence of other systemic manifestations was significantly associated with a greater decline in DLCO%. This suggests that systemic involvement may contribute to impaired gas exchange.

We continued with univariate analyses between PFTs absolute change from baseline and above-mentioned predictors. Regarding the absolute FVC% change, only MMF use barely reached statistical significance with the model explaining 14.1% of the outcome variance. Although not statistically significant, previous MMF use was associated with a greater FVC decline (β=−9.1, 95% CI: −18.8–0.7, p=0.06) while current MMF use showed stability or even improvement (β=2.1, 95% CI: −3.9–8.0, p=0.49).

In the univariate analyses of absolute DLCO changes from baseline, a significant association between other systemic manifestations and absolute DLCO change was noted (β=−10.1, 95% CI: −19.6 – −0.6, p=0.04, R^2^=0.132) indicating worse DLCO outcomes in these patients. A trend was identified with age (β=−0.37, 95% CI: −0.8–0.06, p=0.09, R^2^=0.09), while no significant associations were identified with gender (β= 9.3, 95% CI: −2.0–20.5, p=0.1), CTD-ILD duration (β= −0.6, 95% CI: −2.7–1.5, p=0.59) or MMF (β= 8.7, 95% CI: −1.9–19.3, p=0.15) or rituximab (β= −3.1, 95% CI: −14.5–8.2, p=0.57) use.

In the final multivariate regression model for relative DLCO% change, other systemic manifestations, age and gender were included. The model explained 26.7% of the outcome variance (model p=0.03, R^2^=0.267, adjusted R^2^=0.191). Other systemic manifestations were significantly associated with worse DLCO% change (β=−23.8, 95% CI: −45.8–−1.7, p=0.036) indicating that patients with extrapulmonary manifestations experience a greater decline in DLCO. Older age was also independently associated with worse DLCO% change (β=−0.95 per year, 95% CI: −1.9–−0.04, p=0.041), while sex was not an independent predictor in this model (β= 9.0, 95% CI: −16.2–34.3, p=0.47). For the absolute DLCO difference, four predictors namely age, gender, MMF use and other systemic manifestations were included. The overall model explained 33.2% of the variance in DLCO difference, suggesting a possible effect of these combined factors (model p=0.04) on DLCO decline. While none of the individual values reach statistical significance, age (β= −0.39 per year, 95% CI: −0.84–0.05, p=0.08) and other systemic manifestations (β= −8.8, 95% CI: −19.2–1.5, p=0.09) showed trends toward association with greater DLCO deterioration.

## DISCUSSION

CTD-ILD has been recognised as a significant manifestation of various CTDs, especially SSc, MCTDs, IIMs and RA, carrying a substantial burden on mortality and morbidity. This highlights the need for effective therapies. In this context, following promising data from randomised controlled trials,^6,7^ nintedanib is included in current recommendations for the treatment of patients with CTD-ILD.^5,17^

Our study assesses nintedanib’s effectiveness and tolerability in a real-world setting of Greek patients with CTD-ILD originating from 2 large territorial referral centres. Real-world data include a more heterogeneous patient population, including those with comorbidities often excluded from clinical trials, and, through extended follow-up periods in routine care settings, provide insights into long-term safety and effectiveness of treatments.

In our cohort, PFTs remained relatively stable after nintedanib initiation. In detail, the mean FVC%, showed a non-significant decline which is considered compatible with the results of the autoimmune ILD subgroup of the INBUILD study where FVC% dropped by −2.7 ± 0.9 on nintedanib vs −6 ± 0.8 on the placebo group.^18^

SSc and RA were the predominant CTDs in our cohort, reflecting a distribution comparable to that observed in the INBUILD trial.^18^ Our cohort also includes about 14% of patients with IIM and antisynthetase syndrome, both of which may be associated with severe and refractory to treatment ILD.^19,20^ In this subgroup the administration of nintedanib was also effective, in line with a real-life retrospective study of 36 patients with IIM-ILD, which showed that nintedanib reduced the rate of rapidly progressive ILD.^21^ The Myositis Interstitial Lung Disease Nintedanib Trial (MINT) is currently being conducted to rigorously evaluate the efficacy and safety profile of nintedanib in patients diagnosed with progressive myositis-associated ILD and the results of the study are highly anticipated to contribute valuable insights into the therapeutic potential of nintedanib.^22^

The course of CTD-ILD is variable, ranging from stable disease to rapid progression and respiratory failure.^2^ Several risk factors have been identified that are associated with progression and worse outcomes, depending on the specific CTD. Male sex and older age have been linked to more severe disease in RA-ILD and SSc-ILD.^23–25^ INBUILD subgroup analyses reported no heterogeneity in the effect of nintedanib on slowing FVC decline across age and sex subgroups.^26^ Similarly, in our cohort, only older age showed a slight trend toward worse outcome. It should be noted that the presence of other systemic manifestations consistently emerged as a significant predictor in both relative and absolute DLCO change. This finding can be elucidated by considering the characteristics of a real-world cohort, wherein there was no exclusion of patients presenting with active systemic diseases or confounding comorbidities. The concept that overall immune activation or ongoing systemic inflammation may parallel lung progression has been suggested in various studies. In a prospective cohort study of 215 patients with SSc, the presence of arthritis ever was identified as an independent predictor of ILD progression,^27^ supporting the concept that systemic inflammation is linked to lung function decline. A EUSTAR cohort study showed that baseline synovitis and tendon friction rubs were independently associated with overall disease progression.^28^ Although this study did not exclusively focus on ILD progression, is an important study linking clinical markers of systemic inflammation to SSc progression. Furthermore, a prospective cohort study involving 1419 RA patients reported that higher disease activity was associated with an increased risk of developing RAILD.^29^ A nested case-control study within the German biologic registry RABBIT showed that higher levels of acute inflammatory markers were significantly associated with the occurrence of RA-ILD.^30^

Patients with CTD-ILD receive a range of immunosuppressive therapies targeting both pulmonary involvement and the extrapulmonary manifestations of the underlying CTD. In our cohort, previous MMF use was associated with a greater FVC decline, while current MMF use showed stability or even improvement. This finding could reflect that patients with a history of prior MMF use may have a more severe or progressive disease. Obviously, this does not indicate a direct effect of the medication on FVC decline but rather identifies patients who either failed or didn’t tolerate MMF as a possible subgroup with inherently worse disease trajectories. It should be noted that in a subgroup of the SENSCIS trial, the combination of nintedanib and MMF was associated with a reduced annual rate of decline in FVC compared to placebo, suggesting a potential benefit of this combination in slowing disease progression.^31^ In the management of CTD-ILD, combining the antifibrotic nintedanib with immunosuppressive therapy has been investigated across several real-world observational cohorts with promising results. A retrospective cohort study of 126 patients who started nintedanib for progressive pulmonary fibrosis, while more than two thirds of them were also on prednisolone and a steroid-sparing agent, showed that lung function decline was significantly attenuated after nintedanib initiation.^32^ A Greek single-centre retrospective study involving patients with SSc-ILD found that a triple or dual immunosuppressive/antifibrotic approach with nintedanib plus tocilizumab, frequently alongside MMF or rituximab, was generally well tolerated and PFTs remained stable at 12 months.^33^ It should be noted that combination immunosuppressive therapy in SSc is actively used – particularly for organ involvement like ILD or skin fibrosis – while when compared with autologous hematopoietic stem cell transplantation (AHSCT) in SSc patients eligible for AHSCT the combination of MMF plus rituximab provided similar clinical improvement in skin and lung involvement, but with a better safety profile.^34^ In patients with RA-ILD, a multicentre retrospective study from Spain reported that the combination of nintedanib with corticosteroids and/or various disease modifying antirheumatic drugs (DMARDs) showed no significant decline in mean FVC or DLCO after a median follow-up of 15 months.^35^ Furthermore, the NEREA registry, a multicentre prospective cohort including 150 patients with CTD-ILD, on various immunosuppressives, showed that both nintedanib and pirfenidone lowered functional respiratory impairment risk (defined as ≥ 5% absolute decline in predicted FVC% within a year) compared to no use.^36^ Collectively, these studies along with our cohort, provide evidence supporting the efficacy and safety of combining nintedanib with immunosuppressants in patients with SSc-ILD and broader CTD-ILD.

Our real-world data confirmed that the most common AE are GI events, mainly diarrhoea. In our series, dose reduction occurred in 26.7% while permanent discontinuation occurred only in four patients (6.7%). These numbers are considered lower than those reported in the INBUILD study, where diarrhoea was reported in 63.4% of subjects treated with nintedanib, while permanent discontinuation of the drug was reported in 17.1% of subjects.^18^ This is probably because in everyday care, patients are often pre-emptively counselled on antidiarrhoeal strategies that differ from the rigid protocols of clinical trials. In support of our findings, a German retrospective study of 64 idiopathic pulmonary fibrosis patients reported diarrhoea in only 33% of patients,^37^ far lower than the ≈60% seen in the INPULSIS randomised trials.^38^

Twelve of our patients (20%) died shortly after initiation of the antifibrotic therapy, mainly due to disease exacerbation and infections. The discrepancy between the number of deaths seen in our cohort and those reported in clinical trials may be explained by the fact that real-life cohorts, like ours, include patients with multiple comorbidities (e.g., 23.5% of our patients had PAH and 20.6% had cardiac involvement), that are known predictors of high mortality.^39–41^ Additionally, in our cohort 96.6% of patients received nintedanib as add-on therapy to one or more immunosuppressants, including rituximab alone or in combination with MMF, that had been excluded from clinical trials. In the SENSCIS trial, MMF was allowed in a stable dose, while the IN-BUILD study tested nintedanib monotherapy.^6,7^ Delayed treatment initiation may also affect mortality. Mean duration of ILD in our cohort was about 6 years, while in clinical trials patients who initiated nintedanib long after ILD diagnosis (>80months) had worse outcomes.^42^

It may be beneficial to re-evaluate whether nintedanib treatment is being initiated too late in clinical practice. Our study has certain limitations inherent to its retrospective observational design. These include selection bias, as all non-SSc patients had experienced ILD worsening before nintedanib initiation and only those attending regular follow-up visits had available data, which may limit the generalisability of the findings to those less engaged with the healthcare system. Additionally, the lack of a control group restricts causal inference. We acknowledge that not all potential confounding factors could be accounted for, and the present analysis did not include data on recent medication dose changes or comorbidities which could have influenced the results. As with any retrospective study, missing data were unavoidable and handled by complete case analysis which may introduce bias if the data were missing not at random. To reduce information and measurement bias, all data was extracted in a prespecified form and all PFTs were performed in accredited laboratories. Additionally, multivariate analyses were performed to account for potential confounding. Nevertheless, our study offers valuable insights into the real-world management of CTD-ILD, which is important for understanding how treatments perform in diverse patient populations outside the controlled environment of clinical trials.

## CONCLUSION

Our real-world data across a broad spectrum of CTDILD suggests that nintedanib is associated with disease stabilisation, and it is generally well tolerated. It could be beneficial in combination with immunosuppressives in slowing the rate of lung function decline.

## FUNDING SOURCES

This research did not receive any specific grant from funding agencies in the public, commercial, or not-for-profit sectors.

## DISCLAIMERS

There are no previous online versions of the manuscript. No part of this manuscript is copied or published elsewhere online. No related congress abstract has been published. No editing support has been used. Data are available from the corresponding author upon reasonable request.

## AUTHOR CONTRIBUTIONS

AG and TS wrote the manuscript. AG and MB performed statistical analyses, created the figures, designed the tables, and drafted the results. NZ, MB, EP, MM collected the data, and created the database for the analyses. MAG, CR, DPB, TD and TS conceived of and supervised the study. All authors have reviewed the manuscript. All authors take full responsibility for the integrity and accuracy of all aspects of the work.

## CONFLICT OF INTEREST

AG declares no conflict of interest; MB declares no conflict of interest; NZ declares no conflict of interest; EP declares no conflict of interest; MM has received honoraria from Boehringer Ingelheim and J&J; CR has received speaking fees from Boehringer-Ingelheim which are unrelated to the current work; GAM has received speaking fees from Boehringer-Ingelheim which are unrelated to the current work; DPB has received honoraria from Boehringer Ingelheim for speaking or chairing engagements at various congresses, as well as travel bursaries to support his participation in these events. Additionally, his department has received research grant funding from the company for educational/research projects unrelated to the topic of this paper; TD reports consultancy fees, speaker fees, honoraria, advisory boards from AbbVie, Amgen, Boehringer Ingelheim, GSK, Genesis Pharma, J&J, Lilly, SOBI, UCB and Pfizer, non- financial support for meeting attendance from AbbVie, Genesis Pharma, Janssen, Elpen, Aenorasis, UCB and Pfizer; TS reports speaker fees, honoraria, advisory boards from AbbVie, Amgen, Boehringer Ingelheim, J&J and Lilly, non- financial support for meeting attendance from AbbVie, Amgen, ELPEN, Janssen, Pfizer, and UCB.

## References

[1] Joy GM Arbiv OA Wong CK Lok SD Adderley NA Dobosz KM Prevalence, imaging patterns and risk factors of interstitial lung disease in connective tissue disease: a systematic review and meta-analysis. Eur Respir Rev 2023 Mar 8;32(167):220210. 36889782 10.1183/16000617.0210-2022 PMC10032591

[2] Hilberg O Hoffmann-Vold AM Smith V Bouros D Kilpeläinen M Guiot J Epidemiology of interstitial lung diseases and their progressive-fibrosing behaviour in six European countries. ERJ Open Res 2022 Jan 24;8(1):00597–2021. 35083316 10.1183/23120541.00597-2021 PMC8784757

[3] Jeganathan N Sathananthan M. Connective Tissue Disease-Related Interstitial Lung Disease: Prevalence, Patterns, Predictors, Prognosis, and Treatment. Lung 2020 Oct;198(5):735–59. 32780179 10.1007/s00408-020-00383-w

[4] Guiot J Miedema J Cordeiro A De Vries-Bouwstra JK Dimitroulas T Søndergaard K Practical guidance for the early recognition and follow-up of patients with connective tissue disease-related interstitial lung disease. Autoimmun Rev 2024 Jun;23(6):103582. 39074630 10.1016/j.autrev.2024.103582

[5] Johnson SR Bernstein EJ Bolster MB Chung JH Danoff SK George MD 2023 American College of Rheumatology (ACR)/American College of Chest Physicians (CHEST) Guideline for the Treatment of Interstitial Lung Disease in People with Systemic Autoimmune Rheumatic Diseases. Arthritis Rheumatol 2024 Aug;76(8):1182–200. 38978310 10.1002/art.42861 PMC12646471

[6] Flaherty KR Wells AU Cottin V Devaraj A Walsh SLF Inoue Y Nintedanib in Progressive Fibrosing Interstitial Lung Diseases. N Engl J Med. 2019 Oct 31;381(18):1718–27. 31566307 10.1056/NEJMoa1908681

[7] Distler O Highland KB Gahlemann M Azuma A Fischer A Mayes MD Nintedanib for Systemic Sclerosis-Associated Interstitial Lung Disease. N Engl J Med 2019 Jun 27;380(26):2518–28. 31112379 10.1056/NEJMoa1903076

[8] Wollin L Wex E Pautsch A Schnapp G Hostettler KE Stowasser S Mode of action of nintedanib in the treatment of idiopathic pulmonary fibrosis. Eur Respir J 2015 May;45(5):1434–45. 25745043 10.1183/09031936.00174914 PMC4416110

[9] Wollin L Maillet I Quesniaux V Holweg A Ryffel B. Antifibrotic and anti-inflammatory activity of the tyrosine kinase inhibitor nintedanib in experimental models of lung fibrosis. J Pharmacol Exp Ther 2014 May;349(2):209–20. 24556663 10.1124/jpet.113.208223

[10] Rangarajan S Kurundkar A Kurundkar D Bernard K Sanders YY Ding Q Novel Mechanisms for the Antifibrotic Action of Nintedanib. Am J Respir Cell Mol Biol 2016 Jan;54(1):51–9. 26072676 10.1165/rcmb.2014-0445OC PMC4742925

[11] Mısırcı S Ekin A Yağız B Coşkun BN Başıbüyük F Birlik AM Treatment with nintedanib is as effective and safe in patients with other connective tissue diseases (CTDs)-interstitial lung disease (ILD) as in patients with systemic sclerosis-ILD: A multicenter retrospective study. Clin Rheumatol 2025 Mar;44(3):1187–95. 39920562 10.1007/s10067-025-07323-0 PMC11865171

[12] Barešić M Novak S Perković D Karanović B Mirić F Radić M Real world experience with nintedanib in connective tissue disease-related interstitial lung disease: a retrospective cohort study. Clin Rheumatol 2023 Oct;42(10):2897–903. 37393200 10.1007/s10067-023-06689-3

[13] van den Hoogen F Khanna D Fransen J Johnson SR Baron M Tyndall A 2013 Classification Criteria for Systemic Sclerosis: An American College of Rheumatology/European League Against Rheumatism Collaborative Initiative. Ann Rheum Dis 2013 Nov;72(11):1747–55. 24092682 10.1136/annrheumdis-2013-204424

[14] Aletaha D Neogi T Silman AJ Funovits J Felson DT Bingham CO 3rd 2010 rheumatoid arthritis classification criteria: an American College of Rheumatology/European League Against Rheumatism collaborative initiative. Arthritis Rheum 2010 Sep;62(9):2569–81. 20872595 10.1002/art.27584

[15] Lundberg IE Tjärnlund A Bottai M Werth VP Pilkington C Visser M 2017 European League Against Rheumatism/American College of Rheumatology classification criteria for adult and juvenile idiopathic inflammatory myopathies and their major subgroups. Ann Rheum Dis 2017 Dec;76(12):1955–64. 29079590 10.1136/annrheumdis-2017-211468 PMC5736307

[16] Stanojevic S Kaminsky DA Miller MR Thompson B Aliverti A Barjaktarevic I ERS/ATS technical standard on interpretive strategies for routine lung function tests. Eur Respir J 2022 Jul 13;60(1):2101499. 34949706 10.1183/13993003.01499-2021

[17] Del Galdo F Lescoat A Conaghan PG Bertoldo E Čolić J Santiago T EULAR recommendations for the treatment of systemic sclerosis: 2023 update. Ann Rheum Dis 2025 Jan;84(1):29–40. 39874231 10.1136/ard-2024-226430

[18] Matteson EL Kelly C Distler JHW Hoffmann-Vold AM Seibold JR Mittoo S Nintedanib in Patients With Autoimmune Disease-Related Progressive Fibrosing Interstitial Lung Diseases: Subgroup Analysis of the INBUILD Trial. Arthritis Rheumatol 2022 Jun;74(6):1039–47. 35199968 10.1002/art.42075 PMC9321107

[19] Yamaguchi K Sullivan DI Khushboo S Saygin D Laverde SM Moghadam-Kia S Long-term clinical prognosis of anti-aminoacyl-tRNA synthetase antibodies and interstitial lung disease. Clin Rheumatol 2025 Aug;44(8):3341–52. 40571884 10.1007/s10067-025-07521-w

[20] Jablonski R Bhorade S Strek ME Dematte J. Recognition and Management of Myositis-Associated Rapidly Progressive Interstitial Lung Disease. Chest 2020 Jul;158(1):252–63. 32059958 10.1016/j.chest.2020.01.033

[21] Liang J Cao H Yang Y Ke Y Yu Y Sun C Efficacy and Tolerability of Nintedanib in Idiopathic-Inflammatory-Myopathy-Related Interstitial Lung Disease: A Pilot Study. Front Med (Lausanne) 2021 Feb 3;8:626953. 33614683 10.3389/fmed.2021.626953 PMC7886679

[22] Aggarwal R Oddis CV Sullivan DI Moghadam-Kia S Saygin D Kass DJ Design of a randomised controlled hybrid trial of nintedanib in patients with progressive myositis-associated interstitial lung disease. BMC Pulm Med 2024 Oct 30;24(1):544. 39478532 10.1186/s12890-024-03314-0 PMC11526615

[23] Chan C Ryerson CJ Dunne JV Wilcox PG. Demographic and clinical predictors of progression and mortality in connective tissue disease-associated interstitial lung disease: a retrospective cohort study. BMC Pulm Med 2019 Oct 31;19(1):192. 31672127 10.1186/s12890-019-0943-2 PMC6824100

[24] Qiu M Jiang J Nian X Wang Y Yu P Song J Factors associated with mortality in rheumatoid arthritis-associated interstitial lung disease: a systematic review and meta-analysis. Respir Res 2021 Oct 11;22(1):264. 34635095 10.1186/s12931-021-01856-z PMC8504109

[25] Volkmann ER Tashkin DP Silver R Bostwick CF Assassi S Frost DB Sex differences in clinical outcomes and biological profiles in systemic sclerosis-associated interstitial lung disease: a post-hoc analysis of two randomised controlled trials. Lancet Rheumatol 2022 Oct;4(10):e668–e678. 37745675 10.1016/s2665-9913(22)00193-x PMC10518185

[26] Kolb M Flaherty KR Silva RS Prasse A Vancheri C Mueller H Effect of Nintedanib in Patients with Progressive Pulmonary Fibrosis in Subgroups with Differing Baseline Characteristics. Adv Ther 2023 Dec;40(12):5536–46. 37751022 10.1007/s12325-023-02668-x PMC10611817

[27] Wu W Jordan S Becker MO Dobrota R Maurer B Fretheim H Prediction of progression of interstitial lung disease in patients with systemic sclerosis: the SPAR model. Ann Rheum Dis 2018 Sep;77(9):1326–32. 29875097 10.1136/annrheumdis-2018-213201

[28] Avouac J Walker UA Hachulla E Riemekasten G Cuomo G Carreira PE Joint and tendon involvement predict disease progression in systemic sclerosis: a EUSTAR prospective study. Ann Rheum Dis 2016 Jan;75(1):103–9. 25165035 10.1136/annrheumdis-2014-205295

[29] Sparks JA He X Huang J Fletcher EA Zaccardelli A Friedlander HM Rheumatoid Arthritis Disease Activity Predicting Incident Clinically Apparent Rheumatoid Arthritis-Associated Interstitial Lung Disease: A Prospective Cohort Study. Arthritis Rheumatol 2019 Sep;71(9):1472–82. 30951251 10.1002/art.40904 PMC6716994

[30] Ramien R Rudi T Alten R Krause A Schneider M Schaefer M Impact of systemic inflammation and disease activity on the incidence of interstitial lung disease in patients with rheumatoid arthritis - a nested case-control study within the German biologics register RABBIT. Arthritis Res Ther 2024 Dec 5;26(1):209. 39639378 10.1186/s13075-024-03449-9 PMC11619653

[31] Highland KB Distler O Kuwana M Allanore Y Assassi S Azuma A Efficacy and safety of nintedanib in patients with systemic sclerosis-associated interstitial lung disease treated with mycophenolate: a subgroup analysis of the SENSCIS trial. Lancet Respir Med 2021 Jan;9(1):96–106. 33412120 10.1016/S2213-2600(20)30330-1

[32] Raman L Stewart I Barratt SL Chua F Chaudhuri N Crawshaw A Nintedanib for non-IPF progressive pulmonary fibrosis: 12-month outcome data from a real-world multicentre observational study. ERJ Open Res 2023 Mar 20;9(2):00423–2022. 10.1183/23120541.00423-2022 PMC10026008 36949962

[33] Panopoulos S Tzilas V Bournia VK Tektonidou MG Sfikakis PP. Tocilizumab plus Nintedanib for progressive interstitial lung disease in systemic sclerosis: a one-year observational study. Rheumatol Int 2024 Oct;44(10):1959–66. 39180531 10.1007/s00296-024-05695-1

[34] Keret S Henig I Zuckerman T Kaly L Shouval A Awisat A Outcomes in progressive systemic sclerosis treated with autologous hematopoietic stem cell transplantation compared with combination therapy. Rheumatology (Oxford) 2024 May 3;63(6):1534–8. 37672021 10.1093/rheumatology/kead457

[35] Atienza-Mateo B Serrano-Combarro A Loarce Martos J Vegas-Revenga N Martín López M Castañeda S Real-world evidence of the antifibrotic nintedanib in rheumatoid arthritis-interstitial lung disease. National multicenter study of 74 patients. Semin Arthritis Rheum 2025 Jun;72:152710. 40117729 10.1016/j.semarthrit.2025.152710

[36] Nieto MA Vadillo C Pernaute OS Romero-Bueno F Rodriguez-Nieto MJ Laporta R Role of antifibrotics in progressive pulmonary fibrosis associated to autoimmune diseases: multicenter study from NEREA registry. Respir Res 2025 Jul 2;26(1):234. 40604872 10.1186/s12931-025-03311-9 PMC12225376

[37] Brunnemer E Wälscher J Tenenbaum S Hausmanns J Schulze K Seiter M Real-World Experience with Nintedanib in Patients with Idiopathic Pulmonary Fibrosis. Respiration 2018;95(5):301–9. 29490307 10.1159/000485933 PMC5985741

[38] Richeldi L du Bois RM Raghu G Azuma A Brown KK Costabel U Efficacy and safety of nintedanib in idiopathic pulmonary fibrosis. N Engl J Med 2014 May 29;370(22):2071–82. 24836310 10.1056/NEJMoa1402584

[39] Michelfelder M Becker M Riedlinger A Siegert E Drömann D Yu X Interstitial lung disease increases mortality in systemic sclerosis patients with pulmonary arterial hypertension without affecting hemodynamics and exercise capacity. Clin Rheumatol 2017 Feb;36(2):381–90. 28028682 10.1007/s10067-016-3504-6

[40] Költő G Faludi R Aradi D Bartos B Kumánovics G Minier T Impact of cardiac involvement on the risk of mortality among patients with systemic sclerosis: a 5-year follow-up of a single-center cohort. Clin Rheumatol 2014 Feb;33(2):197–205. 23942767 10.1007/s10067-013-2358-4

[41] Launay D Humbert M Berezne A Cottin V Allanore Y Couderc LJ Clinical characteristics and survival in systemic sclerosis-related pulmonary hypertension associated with interstitial lung disease. Chest 2011 Oct;140(4):1016–24. 21474572 10.1378/chest.10-2473

[42] Kokubu H Takeuchi S Tozawa T Hisada S Yamada Y Itoh Y Assessing prognostic factors correlating with response to nintedanib for connective tissue disease-associated interstitial lung disease: A real-world single-center study. Int J Rheum Dis 2023 Apr;26(4):682–8. 36808836 10.1111/1756-185X.14611

